# Economic and Social Costs of Noma: Design and Application of an Estimation Model to Niger and Burkina Faso

**DOI:** 10.3390/tropicalmed7070119

**Published:** 2022-06-28

**Authors:** Emmanuel Kabengele Mpinga, Margaret Leila Srour, Marie-Solène Adamou Moussa, Marc Dupuis, Moubassira Kagoné, Maïna Sani Malam Grema, Ngoyi-Bukonda Zacharie, Denise Baratti-Mayer

**Affiliations:** 1 Institute of Global Health, Faculty of Medicine, University of Geneva, 1202 Geneva, Switzerland; marie-solene.adamoumoussa@unige.ch (M.-S.A.M.); denise.baratti@unige.ch (D.B.-M.); 2 Health Frontiers, Vientiane, Laos; leila@butterflychildren.org; 3 Institute of Higher Education and Research in Healthcare, Lausanne University Hospital and University of Lausanne, 1011 Lausanne, Switzerland; marc.dupuis@chuv.ch; 4 Centre de Recherche en Santé de Nouna, National Institute of Public Health, Ouagadougou, Burkina Faso; kmoubache@yahoo.fr; 5 Faculté des Lettres et Sciences Humaines, University Abdou Moumouni of Niamey, Niamey, Niger; sanigrema@gmail.com; 6 Department of Public Health Sciences, Wichita State University, Wichita, KS 67260, USA; ngoyi.bukonda@wichita.edu; 7 Faculté des Sciences de la Santé, Université Pédagogique Nationale, Kinshasa-Ngaliema, Democratic Republic of the Congo

**Keywords:** noma, economic, social, cost, burden, Burkina Faso, Niger, estimating model

## Abstract

Background: While noma affects hundreds of thousands of children every year, taking their lives, disfiguring them and leaving them permanently disabled, the economic and social costs of the disease have not been previously estimated. An understanding of the nature and levels of these costs is much needed to formulate and implement strategies for the prevention and control of this disease, or to mitigate its burden. The objectives of our study were to develop a model for estimating the economic and social costs of noma and to provide estimates by applying this model to the specific contexts of two countries in the “noma belt”, namely Burkina Faso and Niger. Methods: Three main approaches were used. The estimation of prevalence levels of potential noma cases and of cases that should receive and actually do receive medical care was carried out using a literature review. The documentary approach made it possible to estimate the direct costs of noma by analyzing the database of a non-governmental organization operating in this field and present in both countries. Indirect costs were estimated using the human capital method and the cost component analysis technique. Results: The direct costs of care and management of noma survivors amount to approximately USD 30 million per year in Burkina Faso, compared to approximately USD 31 million in Niger. They mainly include costs for medical treatment, surgery, hospital stays, physiological care, psychological care, social assistance, schooling, vocational training and care abroad. Indirect costs are estimated at around 20 million in lost production costs in Burkina and around 16 million in Niger. Costs related to premature deaths are estimated at more than USD 3.5 billion in Burkina Faso and USD 3 billion in Niger. Finally, the costs to survivors who are unable to marry are around USD 13.4 million in Burkina and around USD 15 million in Niger. Intangible costs were not calculated. Conclusions: The neglect of noma and inaction in terms of prevention and control of the disease have enormous economic and social costs for households, communities and states. Future studies of this kind are necessary and useful to raise awareness and eradicate this disease, which impacts the health and well-being of children and results in lifelong suffering and severe economic and social costs to survivors and their families.

## 1. Introduction

With an estimated annual incidence of 140,000 cases worldwide, noma, more than any other disease, is a condition that, through its physical, psychological, and social repercussions, calls into question the capacity of societies and communities to protect children and young people from the threats associated with hunger, violence, and social inequality [1,2,3,4]. However, this severity contrasts with the dearth of research into the disease. Acute noma is deadly in 8o% of cases and requires intensive treatment. Survivors will require surgical treatment, which is difficult, expensive and not always affordable. However, there is a lack of prevention at a local level where noma is prevalent, contributing to its high mortality and morbidity.

Research to date has focused on the epidemiological, aetiological and clinical aspects, in particular reconstructive surgery and, more recently, on the prevention of the disease [1,5,6]. There are very few studies, if any, into the social and cultural dimensions associated with determining the occurrence and development of the condition. This gap is indicative of a need to catch up with the increasing view of the importance of social determinants, particularly in poor countries such as Niger and Burkina Faso where a range of prevailing and not-yet explored social determinants may be furtively unfolding and posing a real challenge for public health and health care initiatives. The questions to be addressed here include the rank and position of the mother in polygamous households, her access to means of production, in particular land specifically for widows, and the overall administration of property directly affecting access to and distribution of food. Others point out that the possibility of a link between the disease and the social practices surrounding childbirth, for example, has not yet been explored [6].

Similarly, certain social consequences of noma are often mentioned but they have not yet been explored in depth. This is the case when it comes to the social exclusion and discrimination of direct and indirect (family or households members) survivors, for which the typology forms, mechanisms of expression and courses of action have not yet been studied [7,8]. However, noma also has direct and visible, or indirect and less visible consequences, which affect communities as a whole. The morbidity and mortality attributable to noma entail direct and indirect costs related to care or premature death. Caring for sick children immobilizes parents and results in lost working days. The exclusion of survivors, if any, limits their access to education, alongside their psychological burden of stigma and suffering, including barriers to marriage and a normal social life [9]. Indeed, the inability to marry is identified as a major part of the burden of noma in Laos [10], and is an important reason to request surgery for noma African victims [11].

To date, this entire set of economic and social costs associated with noma has not been the subject of specific studies. With a greater orientation towards political economics, the only publication linking noma to economics is a call for this disease to be used as an economic indicator of the “poverty” of the states where it occurs [12]. Similarly, and at a micro-level, some argue that Noma is a biological indicator of extreme poverty [13].

Understanding the economic and social costs of noma raises a number of interesting issues. Firstly, it complements the arguments on which current control strategies are based. The only public health arguments generally highlighted are related to the burden of morbidity and mortality, and moral arguments are based on the fragility of children and society’s responsibility to give them the protection they need for their development or based on the rights of the child, on human rights and therefore on the obligations of states. However, those arguments do not seem to have produced the desired effects [8,9,14,15]. Both economic and social consequences of noma play an important role in its burden. They have their own consequences on noma victims, for instance, in terms of the ability to get support and pay for medical treatment. Not only noma but also its consequences represent a major public health concern.

Secondly, better knowledge of the costs of noma could also provide a strong economic argument for programs to prevent the disease and support rehabilitation activities for survivors. Furthermore, this knowledge is necessary to combat certain religious, cultural or customary practices that severely limit women’s power, their access to the means of production and the administration of their property. These practices generate, maintain, reinforce and replicate the poverty of women and mothers, whereby the primary victims of such poverty are children, and noma is one of the associated diseases [16,17].

Finally, knowledge of these costs would facilitate broader social mobilization of the many local, national and international stakeholders involved in combating noma by widening the sphere of responsibility beyond the health sector. Certain United Nations agencies such as FAO, UNESCO, ILO, sub-regional organizations (ECOWAS) or regional organizations (AFRICAN UNION), or even financial development institutions (African Development Bank and World Bank), which are criticized for their apparent lack of efficacy, would thus find the necessary rationale for their involvement in the prevention and control of noma [18].

Classified by the WHO as DAOC.31 in the International Classification of Diseases ICD-11 [19], noma seems to have a unanimous definition. According to the WHO, it is “a devastating infectious disease that destroys the soft and hard tissues of the oral and para-oral structures”. Health professionals state that noma or cancrum oris is a gangrenous and multifactorial condition that starts in the gums and then spreads to other parts of the face. It is characterized by a very rapid progression towards necrosis of the soft tissues and underlying bone. It usually occurs in malnourished children with poor oral hygiene and whose bodies are weakened by malnutrition or infectious diseases, especially eruptive fevers or malaria [20]. In this study, we assume that the occurrence of noma in a community imposes costs on the community because resources are used to deal with this problem that could have been allocated to other needs. Such costs are called social insofar as they affect households, communities, and states in the short or long term. As with the study on the costs of drugs from Kopp et al., an estimate of the social costs of noma consists of expressing in a monetary unit the total costs of the consequences of noma for society [21].

Technically, the costs of illness or disability are divided into two broad categories according to Rice: (1) basic costs are those resulting directly from the illness and (2) other associated costs including those not related to the illness. Within each category, there are direct and indirect costs. Direct costs are those for which payments are made and indirect costs are those for which resources are lost. Indirect costs include (1) morbidity costs, the value of lost productivity of persons unable to perform their usual activities or perform at full efficiency due to illness; and (2) mortality costs, the value of lost productivity due to premature death, calculated as the present value of future benefits to society [22,23].

Before describing the research methodology used, it is worth presenting the context in which this study was conducted. Institutionally, this work is part of the research project “*Noma, The Neglected Disease, An Interdisciplinary Exploration of Its Realities, Burden and Framing*” carried out at the Institute of Global Health of the University of Geneva in collaboration with a number of academic partners, UN agencies, governmental entities and non-governmental organizations listed in the acknowledgements. It should be noted, however, that the views expressed in this study are not binding on these partners.

Two countries were selected as part of the project’s research and served as a framework for observation, with their social, demographic and economic data being used to estimate the economic and social impact of noma. Both countries are known for having high levels of noma prevalence [20,24,25] and belong to the “noma belt” countries, which also include Chad, Ethiopia, Mali, Mauritania, Nigeria, Senegal and Sudan.

The two countries also have significant overall differences. The mean annual income was USD 790 for Burkina in 2019 compared to USD 590 for Niger in the same year according to World Bank data [26,27]. Some socio-demographic indicators show little difference between the two countries: life expectancy at birth, which was in 2018, 61.2 years for Burkina compared to 62 years for Niger. By way of comparison, life expectancy in Switzerland was 81.9 years for men and 85.6 years for women in 2018/19 [28], and 84.2 years in Japan in 2018 [29]. The development index values of the two countries rank them for 2019 in 182nd place out of 189 with an index of 0.452 for Burkina, and in 189th place out of 189 with an index of 0.394 for Niger, reflecting levels of extreme “poverty” [30].

Another differential characteristic between the two countries relates to their rates of urbanization, which stood at 31% in Burkina compared to 17% in Niger in 2020 [26,27]. Finally, the under-five mortality rate varies between the two countries; it was 92.2-per-1000-live births in Burkina in 2019 compared to 84‰ in Niger in the same year, reflecting a radical change from known trends indicating that the level of mortality has always been higher in Niger than in Burkina (26‰ in Niger compared to 20‰ in 1980) [29].

This situation is due to another common challenge faced by both countries, namely the deterioration of the security situation—more so in Burkina than in Niger—resulting from the actions of several jihadist groups including Boko Haram. Breaking down the number of attacks on the basis of the countries affected by the attacks, the joint report by NSDD-SHUB and ACRST [31] shows that between September 2019 and April 2020, Burkina Faso led with an average of 34 attacks per month, followed by Mali (23), Niger (5) and Chad (3). The comparative data for these two countries are presented in Table 1.

The objectives of this study were to develop a model for estimating the economic and social costs of noma and to provide estimates of these costs by applying this model to the specific contexts of the two countries presented above.

The results of this study should make it possible to (i) provide decision-makers at various levels with basic tools for control strategies and for assigning the necessary resources to preventive measures and to the care and rehabilitation of noma survivors; (ii) incorporate economic and welfare concerns as a new dimension of noma research, prevention and control; and (iii) formulate proposals for better management of noma survivors in relation to the economic and social requirements of their rehabilitation.

## 2. Methods

### 2.1. Model Design and Data Sources

#### 2.1.1. Estimation of Noma Prevalence in Burkina and Niger (Step 1)

The estimation of the number of potential noma cases in these countries was based on (i) the scientific literature produced on the subject between 2003 and 2021 (in particular the work of Farley [32,33,34], Bello [35], Fieger [18], [24,36], Konsem [37], Bonkoungou [38]; and on (ii) expert opinions consulted to determine the incidence of the disease [3,8,18,24,32,34,35].

In our study, the population at risk of noma is deduced from the respective volumes of populations aged 0–29 according to the population structure data of Burkina and Niger in 2018. The determination of the age groups likely to be affected by noma is derived from the estimate by Bello et al. [35]. By applying the annual incidence rate of 6.4/1000 estimated by Fieger et al. [18] to the number of populations at risk, it was possible to determine the number of potential noma cases in these two countries.

Finally, based on the estimate by Srour et al. [9], for whom the percentage of children with noma receiving medical treatment is estimated at 10% (which the authors consider to be largely optimistic), we deduced the numbers of children with noma “receiving care” or who should receive care in Burkina and Niger.

#### 2.1.2. Identification of the Socio-Economic Indicators Used in The Model (Step 2)

The socio-economic indicators used in this model were identified from research on the economic and social costs of health problems characteristic of noma, in particular the analysis of the burden of malnutrition and malaria [39,40,41,42]. We used life expectancy at birth, mean age at diagnosis, mean age at death of children with noma, untreated mortality rate, mean annual income of the population, mean annual cost of schooling, mean age at marriage, mean dowry rate, and mean numbers of children transferred for care abroad.

The socio-demographic data used are found in (i) national sources namely the National Institute of Statistics and Demography of Burkina Faso and its counterpart in Niger, (ii) international sources such as the national offices of the United Nations Development Program (UNDP) and the World Bank.

The data on social and health expenditure for children with noma were found from the activity reports and a database of the Sentinelles Foundation https://www.sentinelles.org/ (accessed on 22 November 2021), which has been developing prevention and care activities related to noma in Burkina Faso and Niger since 1992.

#### 2.1.3. Estimating the Economic and Social Costs of Noma (Step 3)

The direct costs of noma include the social costs (expenditure on schooling, vocational training and social assistance) for children with noma, as well as the direct costs of care, which include the costs of treatment, surgery, hospital stay, psychological management and physiotherapy in the country and the costs of transfer and care abroad.

The costs arising from the loss of production due to the premature deaths of noma victims constitute the indirect costs. They are estimated by considering that the years of life lost as a result of such deaths or disabilities due to noma lead to productivity losses for individuals, households and states, according to the human capital approach [42,43,44]. The costs associated with the impaired ability of noma women survivors to attract marital partners, i.e., their inability to marry or the difficulties they face when it comes to marrying, have been included in these indirect costs.

The costs related to the stigma, discrimination and suffering of noma survivors constitute the category of intangible costs.

## 3. Results

### 3.1. Application of the Estimation Model to Burkina Faso and Niger

**Figure 1:**
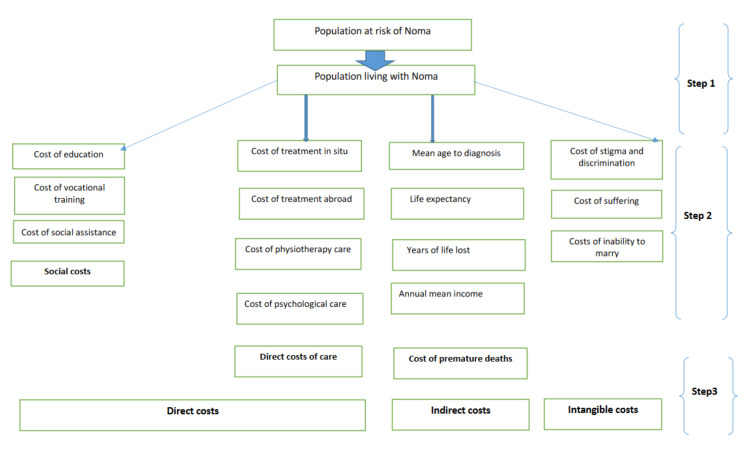
Cost estimation model. Economic and social costs of noma.

Figure 1 below shows the estimation model used and its different components.

#### 3.1.1. Prevalence of Noma

Burkina Faso and Niger had 20,244,080 and 22,442,948 inhabitants, respectively, in 2018. Based on the estimate by Bello et al. [35] that noma mainly affects persons aged 0–30 years, we deduced from the population structure of those countries that the population at risk for noma is 14,939,063 (0–29 years) and 16,540,452 (0–29 years), respectively, in 2018.

From these two estimates, the numbers of potential noma cases were calculated based on the annual incidence rate of 6.4 cases per 1000 people as estimated by Fieger et al. [18] and agreed upon by the experts consulted (Baratti-Mayer, Srour, 2020). These potential case numbers are 95,610 for Burkina Faso and 105,858 for Niger based on the 2018 figures.

Finally, as a third step, we deduced from the latter two estimates the numbers of potential noma cases receiving care or who should receive care, by taking the 10% of potential noma cases based on indications by Srour et al. [9]. These authors have indicated that noma survivors remain hidden and that only 10–15% of survivors seek care. We thus estimated 9561 cases needing care for Burkina Faso compared to 10,585 cases for Niger in 2018. These results are summarized in Table 2 below.

#### 3.1.2. Health and Socio-Demographic Indicators

The levels of life expectancy at birth (2018) used are 61.2 years and 62.0 years for Burkina and Niger, respectively, based on data from the Population Data website [43,44]. The mean age of death from noma is 6 years. This is derived from studies by Bello et al. [35], Idigbe et al. [45], Adeniyi et al. [3], Farley et al. [34], Tall et al. [46], and Konsem et al. [37]. The mean age of noma patients in these studies ranged from 4 to 7.6 years. The years of life lost were calculated by subtracting the life expectancy levels at birth from the mean age at death. In Burkina, it was calculated that 55.2 years of life are lost, compared to 56 years in Niger. The estimated mortality rate without care is 90%. This estimate is based on reports by Bourgeois et al. [4], Srour et al. [8], Zwetyenga et al. [47], Prado-Calleros et al. [48] and the recent scoping review by Farley al. [33].

The mean annual per capita income is USD 750 for Burkina Faso and USD 570 for Niger in 2018, according to World Bank data [26].

These results are summarized in Table 3.

#### 3.1.3. Economic and Social Costs of Noma in Burkina Faso and Niger

The direct costs of care and management of noma survivors calculated on the basis of data provided by the Sentinelles Foundation amount to approximately USD 30 (28,179,901) million per year (2018) in Burkina Faso, compared to approximately USD 31 (30,746,063) million in Niger. They include 48% of care and social assistance costs and 52% of costs related to care abroad for under 200 (172) people in Burkina Faso. The results for Niger are divided along similar lines. The major cost drivers are—in order of importance—hospital or health center stay (USD 4,388,805), medical care (USD 3,298,545), surgery (USD 2,294,800), physiotherapy (USD 1,457,198) and assistance with income-generating activities (USD 1,406,398) in Burkina Faso. This order of importance is almost identical for Niger.

In this category of direct costs, the most important item is the cost of surgical transfers abroad, estimated at USD 85,000 per case in 2020 according to data provided by the Sentinelles Foundation, and, for the 172 and 185 potential transfer cases, these costs are around USD 15 million (14,620,000) and USD 16 million (15,725,000) for Burkina Faso and Niger, respectively.

Calculated based on potential cases of noma in persons of legal working age (15–29 years) and mean annual incomes of USD 750 and USD 570 for the two countries, the indirect costs of lost production are around USD 20 million (19,402,500) for Burkina and over USD 15 million (16,291,170) for Niger for the year of the study.

In this same category, the costs related to premature death (human capital approach) were calculated by taking into account the number of potential noma-related deaths (90%) of potential cases (86,046 and 95,272), the mean annual income (USD 750 and 570) and the years of life saved (55.2 and 56.0 years). The estimates reached are USD 3,562,428,600 for Burkina and USD 3,041,082,240 for Niger, which corresponds to the highest cost by far.

Finally, we calculated the costs of *being unable to marry* by noma women survivors, i.e., their inability to marry or the difficulties they face when it comes to marrying. When women are not able to marry, the absence of dowry can be considered an economic burden for their families. The number of women of marriageable age (15–29) among potential noma cases and the average costs (dowry and related expenses) of marriage estimated at USD 1000 in both countries by two independent informants were taken into account. These costs are therefore USD 13,365,000 in Burkina and USD 14,820,000 in Niger.

The intangible costs of pain, suffering, stigma and discrimination are difficult to estimate and have not been calculated.

These results are presented in detail in Table 4 and Table 5 and summarized in Table 6.

## 4. Discussion

The objective of this study was to calculate the economic and social costs based on a model previously developed and applied to the contexts of two countries in the “noma belt” (Burkina Faso and Niger). Although this is the first study of its kind for this disease, the level of direct costs, particularly those of medical care (treatment, surgery, accommodation, psychotherapy and physiotherapy), averaged USD 1829 per case in both countries, a figure close to the USD 1,791.7 direct costs of care for a case of a condition sometimes confused with noma, Buruli ulcer, in Benin [51]. This same level does not seem to be fundamentally different from the 47 euros per week (2280 euros/year) of care costs for a noma case detected and treated in Togo, the neighboring country of Benin, Niger and Burkina [52].

The report on mean annual income does not include the significant disparities across these countries. Families living in extreme poverty have never seen this annual income. Their lack of income is not reflected in the mean annual per capita income. Their level of poverty is often not recorded.

The estimated cost levels depend not only on known socio-demographic indicators but also on the estimated prevalence of noma cases. However, there are several factors that limit the validity of incidence levels from various sources. Firstly, incidence rates vary enormously between studies; from 8.3 per 100,000 persons at risk in Northwest Nigeria to 7 per 1000 children aged 1–16 years in the same country [35,52].

Next, the 1998 WHO estimate of 140,000 new cases of noma per year seems to have been greatly exceeded more than a quarter of a century later due to (i) the deterioration of the security situation in the region; (ii) the effects of the 1994 devaluation of the West African CFA franc on the standard of living of the population; (iii) and the consequences of climate change on agricultural production. Associated with noma, the rates of acute malnutrition, chronic malnutrition and low body weight at a national level were 9.1% (of which 1.0% in the severe form); 24.9% and 17.6% in December 2020, and only 21.9% of children in Burkina Faso have a minimum acceptable diet [53].

Finally, it should be remembered that most studies on the epidemiology of noma are based on hospital data, leading some to suggest that these estimates may not accurately reflect the actual incidence of acute noma or the prevalence of patients with noma sequelae, as they are based on expert opinion or historical data. It is also not known which stages of noma are included in these estimates [4,34]. It is reasonable to suggest that the costs calculated on these bases appear to be clearly underestimated.

Other social and economic costs of noma have not been included in our study. These include pre-hospital care or costs resulting from the use of traditional and alternative medicines. It is known that about 80% of the population in this region uses traditional medicine, and, in Niger, 40% of children suffering from noma were first seen by a traditional practitioner [24]. Results from an unpublished study of survivors’ experiences in Niger indicate that the costs of treating noma prior to management by the Sentinelles Foundation ranged from 25,000 to 500,000 XOF (2021), or an average of 118,400 XOF or USD 202.75, equivalent to 35% of the mean annual income in the country, which represents a huge burden on households and probably explains the high level of mortality of children with the disease [54].

In the same vein, the reader will note that the costs of noma prevention programs and activities have not been calculated. This should not be interpreted as a complete absence of such activities in the countries where noma occurs and during the period covered by the study. It is due to the impossibility of identifying such activities at the central, state, or regional level by the various national or international organizations directly involved in the fight against noma or those organizations operating in related areas, such as in the field of nutrition.

In this area, a study reporting on the intervention of Médecins sans Frontières in the prevention of malnutrition in Niger indicates that approximately USD 59 had been distributed to households in addition to food distribution between August and December 2021. This amount is not only recommended by the government but is also practiced by other international organizations [55].

Finally, the funeral costs often borne by households and communities were not included in our study. Nevertheless, they are known to be high and deserve to be incorporated into future estimates. For the two countries considered, total funeral costs following deaths due to poor sanitation were estimated at USD 130 million in 2012 in Niger and USD 150 million in Burkina Faso by the World Bank’s Water and Sanitation Program [56].

Even without including pre-hospital costs, costs of prevention activities or costs of funerals in both countries, the estimated direct costs represent a huge burden for households, communities, states and other local or international stakeholders. The direct costs of caring for noma victims represent about half of the costs of social sector staff in Burkina in 2014—about 39 billion CFA francs—and would cover the mean nationwide health expenditure of one million people in Niger in 2016 [57,58].

The costs of schooling for each child with noma were calculated on the basis of USD 175 from the Sentinelles Foundation data. This figure is not so far from the estimate provided by specialist sources in the sector, indicating that the household contribution to pre-school education expenditure would be around USD 140 in Niger in 2020 [58]. Using the estimates of the latter source, these costs would cover one tenth of the Niger government’s total spending on the health sector in 2016.

However, it should be remembered that these costs may be underestimated or overestimated due to a double effect on the prevalence of school-age children with noma, which may be lower in the younger age groups (0–6 years) on the one hand, and in adolescents and adults with noma, who are inside or outside the education system.

The indirect costs are the highest. Those related to lost production, estimated at USD 16 million, represent half of the costs of implementing free healthcare measures, estimated at 20 billion XOF during 2016 in Burkina Faso (i.e., 11.2% of the total budget of the country’s Ministry of Health and one-third of its 2018 investment budget in the same sector) [59]. The costs associated with premature deaths of people with noma represent 62.5% of Niger’s total budget in 2022, estimated at USD 4.86 billion. However, it should be noted that the average annual income used to calculate indirect costs does not include the significant disparities across these countries.

One question that arises at this point is: which institutions or structures should absorb these costs, i.e., who should pay for them? In the case of noma, as with other health problems, it is known that it is the households (i.e., the population, the state and its branches in the context of administrative decentralization, and above all external aid from non-governmental organizations and agencies of the United Nations system) that cover these expenses. On this subject, Kouyate et al. point out that, in Burkina Faso, the state and external aid, respectively, cover 18% and 28% of all health expenditure and that the remaining 54% is financed by the population [58]. These proportions are similar to those derived from Owundi’s analysis of data from the WHO’s 2013 health statistics report, which establishes that, in the cases under review, private expenditure represented 60.4% of total health expenditure in Burkina Faso in 2000, compared to 55.5% in Niger in the same year [58].

In this context, it should be stressed that the (non-funding or) under-funding of prevention, care and rehabilitation activities for noma survivors is an undeniable fact. The denial of the prevalence of this disease by certain states, the stigmatization and discrimination affecting people with the disease and their families, the refusal of care by some parents, ignorance of the disease, including by traditional practitioners and health professionals in the modern medicine sector, and the fact that this disease is not included in the list of neglected tropical diseases constitute the major obstacles to the financing of projects and programs for the care of noma [32,60]. As a result, there are only a few NGOs funding these activities, and only to a very limited extent [9].

This underfinancing or non-financing of the fight against noma leads not only to the levels of costs estimated above but also to additional costs associated with their increase. These are, in effect, the costs of inaction—which may be seen as the overall social and economic costs of the failure to invest in combating this disease. Inaction, or preserving the status quo, impacts individuals and on economic and social structures in the long term. Such costs seem not only unjustified, but also unacceptable.

This inaction is itself a corollary of the recognition of the existence and prevalence of noma in the affected countries, and also of the commitment of the states concerned to address it. This recognition and commitment varies enormously between states. In Africa, where noma was declared a health priority in 2006 [61], some states are strongly committed to fighting the disease, while others have no information on its prevalence or on the need for intervention in this area.

As can be seen, the levels of economic and social costs of noma depend on the economic, social, legal and cultural context of each country on the one hand, and on the state of the health system and security environment on the other. Estimates of these costs should consider these contexts by avoiding a mimetic application of the estimation model presented in this study.

As the first study, to our knowledge, to develop and test a model for estimating the costs of noma in the research into the condition, our study has some obvious strengths. Firstly, the estimation model used is an adaptation of an earlier concept that has proven its validity in estimating the social and economic costs of one of the most serious forms of human rights abuse, namely torture. Secondly, the direct cost values used are actual data from an organization that has been working on noma for many years. The estimated direct costs were therefore closer to those known from the context of medical care for other types of diseases as mentioned above, thus proving the external validity of our results. Finally, the study is based on the authors’ actual knowledge of the contexts of the two countries as well as the support of local networks and expertise used in this research.

However, these strengths cannot hide certain limitations, some of which have already been mentioned. The first limitation concerns the existence of cost categories that were not estimated in our study. These include the costs of preventive activities or those arising from pre-hospital care. The second limitation is that most of the data used come from the database of a single non-governmental organization. These data are a function of the organization of this structure, i.e., its choices, resources and cardinal values in the provision of care and/or social assistance. Third, the estimated costs may increase quickly, and lose representativeness. Finally, the present cost estimates rely on incidence and prevalence estimates from studies with a very small number of cases. Thus, they are indicative but might be underestimated.

Furthermore, the level of costs associated with care abroad may appear high to some. The patient transfers and the care in question took place in Switzerland, which has some of the most expensive living standards and healthcare costs in the world. Switzerland is ranked as the 2nd most expensive country in the world after Bermuda, while France is ranked 19th and Italy 32nd according to the Numbeo Quality of Life Index and other authors [62]. These costs may be overestimated in this case and do not reflect the world average.

Part of this discussion focused on the difficulties of determining the incidence rate of this disease and estimates of prevalence levels for potential populations at risk of noma and of those receiving treatment or who should receive treatment. In the absence of consensus on this issue, studies on the burden of this disease will suffer from this shortcoming.

However, this shortcoming is by no means the only reason why new research on noma in general, and on its economic and social costs in particular, is needed. This field of research is characterized in particular by:

1. An apparent “gap” in research on noma, with the majority of work devoted to West Africa in general and to the countries belonging to the “noma belt” in particular;
2. A predominance of clinical studies in the epidemiology of noma, with a critical lack of population-based studies, even though these are necessary to understand the extent of the condition;
3. An apparent dearth of studies on the role and importance of social structures both in the emergence of noma and in the strategies to combat it;
4. A lack of knowledge of the disease among health professionals, including traditional healers [3,36,63];

In view of this situation, new perspectives for research and action should focus on:

1. Widening the scope of research to include states where prevalence levels are not known and where there are no health data on the subject in existence;
2. Testing the proposed noma cost estimation model by applying it to different contexts and different health and social systems;
3. Orienting cost studies towards understanding the distribution of costs by paying agent (households, state, NGOs);
4. Incorporating social sciences, in particular, political economics, sociology, history, anthropology, political epidemiology and human rights into the research and activities to combat noma;
5. Encouraging and educating states that are already committed, not very committed or still in denial to invest in the prevention and fight against noma, thus avoiding the enormous costs of their inaction;
6. Training health professionals, including traditional practitioners, in the diagnosis and care of people with noma and in the collection, management and use of social and health data on noma cases occurring in the community or received in consultation;
7. Sharing good practices in prevention and care, and in the rehabilitation of noma survivors;
8. Aiming in the long term at completely eradicating this disease of shame and collective irresponsibility towards our children.

## 5. Conclusions

This study aimed to design a model for estimating the economic and social costs of noma and to apply it to the specific contexts of two African countries: Niger and Burkina Faso. These objectives were achieved through efforts to conceptualize and categorize the costs on the one hand, and to identify and collect data on the socio-demographic indicators necessary for this estimation on the other.

The foundations for estimating the economic and social costs of noma have thus been laid. They constitute a call for the continuation of such studies, taking into account the different political, demographic, economic and security contexts, as well as the different health and social systems.

The failure of governments to invest in the prevention and control of noma has and will continue to have enormous costs for households, communities, states and the children themselves in the long term.

Analysis and understanding of both the causes and the structural effects of noma are necessary steps for such investments. Achieving them is our shared responsibility.

## Figures and Tables

**Table 1:** Presentation of the Burkina and Niger context.

Indicators	Burkina Faso	Niger
Surface area in km^2^	270,764	1,266,491
Population (inhabitants)/2019	20,870,060	23,310,179
Density (number of inhabitants per km^2^)	77.08	18.40
Mean annual income USD/2018	750.00	570.00
Life expectancy at birth/2018	61.20	62.00
Mortality rate < age 5 in ‰/2018	92.20	84.00
Urbanization rate in %/2020	31.00	17.00
Human Development Index/2019	0.452	0.394
HDI rank in 2019	182/189	189/189

Developed on the basis of data from [26,31].

**Table 2:** Prevalence of Noma cases in Burkina Faso and Niger in 2018.

Indicators	Burkina Faso	Niger
Total population	20,244,080	22,442,948
Population at risk of noma	14,939,063	16,540,452
Potential noma cases	95,610	105,858
Potential noma cases receiving treatment	9561	10,585

**Table 3:** Socio-economic indicators for Burkina Faso and Niger in 2018.

Indicators	Burkina Faso	Niger	Sources
Life expectancy at birth (years)	61.20	62.00	[43,44]
Mean age of mortality from noma (years)	6.00	6.00	[35,49,50]
Years of life lost	55.20	56.00	[35,48]
Mortality rate (without treatment) in %	90.00	90.00	[8,47]
Mean annual income per inhabitant in USD	750.00	570.00	[26,27]

**Table 4:** Economic and social costs of noma in Burkina Faso in 2018.

Cost Category	Population	Mean Cost per Person in USD	Total Costs in USD	Data Sources
*1. Direct costs*	9561		28,179,901	
Costs of treatment	9561	345	3,298,545	A.
Costs of surgery	5737	400	2,294,800	B1.
Costs of accommodation	5737	765	4,388,805	B2.
Costs of psychological management	5737	65	372,905	B3.
Costs of physiotherapy	5737	254	1,457,198	B4.
Costs of assist. W. Income-gen. Activities	5737	245	1,406,398	B5.
Costs of schooling/vocational training	1950	175	341,250	B6.
Costs of care abroad	172	85,000	14,620,000	B7.
*2. Indirect costs*				
Costs of loss of production	25,870	750	19,402,500	C1.
Costs related to premature death	86,049	750 × 55.2	3,562,428,600	C2.
Costs of inability to marry	13,356	1000	13,365,000	C3.
*3. Intangible costs*	NN	NN	NN	

A. Tall et al. [20]: The mean cost was 255,000 FCFA in 2001. We converted this sum to US dollars at the 2001 rate (345). B1. Sentinelles Foundation database (2020) and [8]: Mean cost per case operated (CHF 393) converted to USD 2020 (400). B2. Sentinelles Foundation database (2020). Mean cost of medical care, (CHF 752) converted to USD 2020 (765). B3. Sentinelles Foundation database (2020). Mean cost of medical care, (CHF 58) converted to USD 2020 (65). B4. Sentinelles Foundation database (2020). Mean cost of medical care, (CHF 64) converted to USD 2020 (254). B5. Sentinelles Foundation database (2020) and expert estimate. Mean cost of assistance (raising livestock, agriculture, trade (CHF 233) converted to USD 2020 (245). B6. Sentinelles Foundation database (2020). Mean cost of schooling (CHF 163) converted to USD 2020 (175). B7. Sentinelles Foundation database (2020). Mean cost of transfer and care, including all charges (CHF 77,850) converted to USD 2020 (85,000). C1. World Bank [26] and potential noma cases of legal working age 15–29 years C2. Death of 90% of potential noma cases without treatment. C3. Women 15–29; 2,820,222, population at risk of noma: 2,086,964, potential noma cases among women 13,356.

**Table 5:** Economic and social costs of noma in Niger in 2018.

Cost Category	Population	Mean Cost per Person in USD	Total Costs in USD	Data Sources
*1. Direct costs*	10,585		30,746,063	
Costs of treatment	10,585	345	3,651,825	A.
Costs of surgery	6372	400	2,548,800	B1.
Costs of accommodation	6372	765	4,874,580	B2.
Costs of psychological management	6372	65	414,180	B3.
Costs of physiotherapy	6372	254	1,618,488	B4.
Costs of assist. W. Income-gen. activities	6372	245	1,561,140	B5.
Costs of schooling/vocational training.	2166	175	379,050	B6.
Costs of care abroad	185	85,000	15,725,000	B7.
*2. Indirect costs*				
Costs of loss of production	28,581	570	16,291,170	C1.
Costs related to premature death	95,272	570 × 56	3,041,082,240	C2.
Costs of inability to marry	14,820	1000	14,820,000	C3.
*3. Intangible costs*	NN	NN	NN	

A. Tall et al. [20]: The mean cost was 255,000 FCFA in 2001. We converted this sum to US dollars at the 2001 rate (345). B1. Sentinelles Foundation database (2020) and [8]: Mean cost per case operated (CHF 393) converted to USD 2020 (400). B2. Sentinelles Foundation database (2020). Mean cost of medical care, (CHF 752) converted to USD 2020 (765). B3. Sentinelles Foundation database (2020). Mean cost of medical care, (CHF 58) converted to USD 2020 (65). B4. Sentinelles Foundation database (2020). Mean cost of medical care, (CHF 64) converted to USD 2020 (254). B5. Sentinelles Foundation database (2020) and expert estimate. Mean cost of assistance (raising livestock, agriculture, trade (CHF 233) converted to USD 2020 (245). B6. Sentinelles Foundation database (2020). Mean cost of schooling (CHF 163) converted to USD 2020 (175). B7 Sentinelles Foundation database (2020). Mean cost of transfer and care, including all charges (CHF 77,850) converted to USD 2020 (85,000). C1. World Bank [26] and potential noma cases of legal working age 15–29 years. C2. Death of 90% of potential noma cases without treatment. C3. Women 15–29; 2,820,222, population at risk of noma: 2,086,964, potential noma cases among women 13,356.

**Table 6:** Summary of economic and social costs of noma in Burkina Faso and Niger.

Cost Categories	Burkina Faso Populations/Costs in US Dollars		Niger Populations/Costs in US Dollars	
*1. Direct costs*	9561	28,179,901	10,585	30,746,063
Costs of treatment	9561	3,298,545	10,585	3,651,825
Costs of surgery	5737	2,294,800	6372	2,548,800
Costs of accommodation	5737	4,388,805	6372	4,874,580
Costs of psychological care	5737	372,905	6372	414,180
Costs of physiotherapy	5737	1,457,198	6372	1,618,488
Costs of schooling/vocational training	1950	341,250	2166	379,050
Costs of assist. W. Income-gen. Activities	5737	1,406,398	6372	1,561,140
Costs of care abroad	172	14,620,000	185	15,725,000
*2. Indirect costs*				
Costs of loss of production	28,581	19,402,500	28,581	16,291,170
Costs related to premature death	95,272	3,562,428,600	95,272	3,041,082,240
Costs of inability to marry	14,820	13,365,000	14,820	14,820,000
*3. Intangible costs*	NN	NN	NN	NN

Developed by us on the basis of data from Table 3 and Table 4.

## Data Availability

Some of the data mainly used in this article come from the Sentinelles Foundation database sheet. Any request to use these data should be made with the agreement of this Foundation.

## Appendix A

List of Funders and Partners of the Noma Project:

1. Lead Organizations are the University of Geneva, Switzerland; The University of York, UK; The Swiss Tropical and Public Health Institute, Basel, Switzerland.
2. Funders Organisations are Swiss Network for International Studies, Geneva, Switzerland; Hilfsaktion Noma e.V. Regensburg, Germany; Service de la Solidarité Internationale- Geneva, Switzerland; Noma-Hilfe-Schweiz, Zurich–Switzerland; Winds of Hope, Lausanne, Switzerland.
3. Partners Organisations are Foundation Sentinelles, Lausanne, Switzerland; Hilfsaktion Noma e.V. Regensburg, Germany; Health Frontiers Laos, Vientiane, Laos; Médecins Sans Frontières, Geneva, Switzerland; SongES, Niamey, Niger; International No Noma Federation, Lausanne, Switzerland.
4. Academics Partners are the Centre Interfacultaire en Droits de l’Enfant, University of Geneva, Switzerland; Centre de recherche en santé, Burkina Faso; Geneva Health Forum, Geneva, Switzerland.
5. Governmental Bodies are the Ministry of Health, Ouagadougou, Burkina Faso
6. Programme National de lutte contre les maladies bucco-dentaires et le noma, Niamey, Niger.
7. Intergovernmental Partners are the United Nations Human Rights Council Advisory Committee; the United Nations Children’s Fund (UNICEF), Niger; the World Health Organisation, Geneva.
8. Special Thanks to Valérie Elsig from Sentinelles Foundation for her support to this study.
